# Targeting G-quadruplexes with Organic Dyes: Chelerythrine–DNA Binding Elucidated by Combining Molecular Modeling and Optical Spectroscopy

**DOI:** 10.3390/antiox8100472

**Published:** 2019-10-10

**Authors:** Alessio Terenzi, Hugo Gattuso, Angelo Spinello, Bernhard K. Keppler, Christophe Chipot, François Dehez, Giampaolo Barone, Antonio Monari

**Affiliations:** 1 Institute of Inorganic Chemistry, University of Vienna, Währingerstrasse 42, A-1090 Vienna, Austria; bernhard.keppler@univie.ac.at; 2 Donostia International Physics Center, Paseo Manuel de Lardizabal 4, 20018 Donostia, Spain; 3 Université de Lorraine and CNRS, LPCT UMR 7019, F54000 Nancy, France; hfbm.gattuso@gmail.com (H.G.); Christophe.Chipot@univ-lorraine.fr (C.C.); francois.dehez@univ-lorraine.fr (F.D.); 4 CNR-IOM DEMOCRITOS c/o International School for Advanced Studies (SISSA), 34136 Trieste, Italy; aspinello@sissa.it; 5 Laboratoire International Associé Centre National de la Recherche Scientifique et University of Illinois at Urbana−Champaign, Urbana, IL 61820, USA; 6 Department of Physics, University of Illinois at Urbana−Champaign, 1110 West Green Street, Urbana, IL 61801, USA; 7 Dipartimento di Scienze Biologiche, Chimiche e Farmaceutiche, Università di Palermo, Viale delle Scienze, 90128 Palermo, Italy

**Keywords:** guanine quadruplexes, anticancer drugs, all atom molecular dynamics, circular dichroism

## Abstract

The DNA-binding of the natural benzophenanthridine alkaloid chelerythrine (CHE) has been assessed by combining molecular modeling and optical absorption spectroscopy. Specifically, both double-helical (B-DNA) and G-quadruplex sequences—representative of different topologies and possessing biological relevance, such as telomeric or regulatory sequences—have been considered. An original multiscale protocol, making use of molecular dynamics (MD) simulations and quantum mechanics/molecular mechanics (QM/MM) calculations, allowed us to compare the theoretical and experimental circular dichroism spectra of the different DNA topologies, readily providing atomic-level details of the CHE–DNA binding modes. The binding selectivity towards G-quadruplexes is confirmed by both experimental and theoretical determination of the binding free energies. Overall, our mixed computational and experimental approach is able to shed light on the interaction of small molecules with different DNA conformations. In particular, CHE may be seen as the building block of promising drug candidates specifically targeting G-quadruplexes for both antitumoral and antiviral purposes.

## 1. Introduction

The biological role of DNA is fundamental in storing and preserving the genetic information of living organisms and in ensuring its faithful replication. As such, any deregulation in the genome integrity or in the replication of the genetic information may result in extremely serious consequences, for example leading to cell death or to spurious mutations, and in the case of eukaryotic organisms, to carcinogenesis [1,2,3,4,5,6,7]. Hence, the interaction of small molecules, such as pollutants, with DNA can be seen as a serious environmental and societal threat [8,9,10,11]. One of the best known examples are polyaromatic hydrocarbon compounds [12,13,14], recognized as carcinogenic agents, the interaction of which with UltraViolet A (UVA) light [15,16,17] may strongly enhance the toxicity of the DNA exposome [18]. However, DNA sensitization by small molecules can also be exploited as a therapeutic strategy against severe diseases such as cancer. Targeting DNA in cancer chemotherapy has, indeed, been pursued for a long time, both exploiting direct coordination with metal compounds, such as cisplatin, or noncovalent interaction with organic and organometallic small molecules, including ruthenium complexes [19,20,21,22], bleomycin [23], and doxorubicin [24]. Although this strategy has proven to be extremely successful in the treatment of a number of cancers [25], it is seriously hampered by unwanted side effects. Indeed, many DNA targeting drugs dramatically lack selectivity, hence producing a series of undesirable systemic effects. As an example, cisplatin [26,27] is totally nonselective against cancer or healthy cells, with the only partial selectivity being caused by the increased duplication rate of cancer cell lines. As a consequence, severe secondary effects such as immunodeficiency or kidney toxicity are usually listed. One way to increase selectivity is sometimes achieved through photosensitization and photodynamic therapy (PDT) [28,29,30] (i.e., the combination of a DNA targeting drug that is usually inactive, but is activated through irradiation, thereby allowing its effects to be localized in specific body areas). Although promising, PDT still suffers from important drawbacks notably related to its limited light penetration, which restricts its applicability to rather superficial or accessible lesions. Furthermore, and because it is usually performed via activation of singlet oxygen [30,31], its efficacy is strongly reduced for hypoxic solid tumors, although recently some non-oxygen-related light-assisted chemotherapy agents have been reported [32,33]. 

An alternative strategy to achieve greater treatment selectivity is to tackle non-coding DNA regions, in particular those able to fold into G-quadruplexes (G4s). G4s can be formed in single stranded, guanine-rich, self-organizing DNA regions, which are most notably present in telomeres and in gene promoters [34]. G4s are constituted by two or more guanine quartets, locked together by Hoogsteen hydrogen bonds and strong π–π stacking interactions. The stability of the aggregate is further enhanced by the interaction with positive ions, usually Na^+^ or K^+^, inside the electron-rich central channel. The different relative orientation of the glycosidic bond of the tetrads allows the G4 conformation to be classified as parallel, antiparallel, or hybrid [35]. G4 targeting strategy is based on the stabilization of the quadruplex arrangement through the interaction with external sensitizers. Indeed, stabilization of telomeric G4s may induce inhibition of telomerase, an enzyme overexpressed in a number of cancers that is responsible for misregulation of the length of the telomeres, eventually conferring the “immortality” typical of cancer cells [36,37]. On the other hand, stabilization of G4 arrangements in gene-promoting regions may avoid the interaction of the DNA sequence with the transcription factors responsible for the initiation of the gene expression, and may ultimately lead to selective suppression of gene expression [37,38,39]. Very recently, and following the observation of RNA G4s, this strategy has also been suggested for antiviral therapy (e.g., against HIV or Zika) [40]. Owing to the high amount of guanine constituting the G4 sequences, the latter represent obvious hot spots for oxidative lesions induced by external sensitizers and drugs. Important structural rearrangements [41], or even epigenetic related modulations [42] induced by G4 guanine oxidation, have been recently reported.

Even though this potential therapeutic strategy is extremely appealing, rational design of efficient and selective B-DNA and G4 binders is somewhat burdened by the lack of precise structural information on the aggregates, and conversely by the strong polymorphisms evidenced by both native B-DNA and G4. As an example, it has been recently shown that parallel G4s may exhibit a spontaneous transition to their hybrid arrangement in a crowded environment [43,44,45]. Obviously, the interaction with external agents that may shift the conformational equilibrium and exhibit different binding modes strongly complicates the rationalization of the phenomenon [46]. The lack of precise experimental structural determination of DNA–drug complexes also stems from the difficulty of crystallizing aggregates required for X-ray analysis. In addition, the inherently static picture offered by a crystal structure may mask polymorphism or dynamic behavior that completely dominates the physics and chemistry of the aggregates in certain cases. An alternative strategy to obtain structural information about DNA–G4-drug interactions, which is much less burdened by experimental difficulties, relies on exploiting electronic circular dichroism (ECD, or simply CD). Indeed, this spectroscopic technique, based on the fine coupling exhibited by the units of multichromophoric systems, is extremely sensitive to even small structural variations. However, the rich density of information embedded in ECD spectra is usually extremely difficult to extract, leading to a nonoptimal exploitation of the technique itself.

Under these premises, we have recently proposed a novel protocol that combines ECD measurements with ECD simulation, using multiscale hybrid quantum mechanics/molecular mechanics (QM/MM) methods [47,48,49]. Most notably, ECD simulation is performed with a statistic ensemble of snapshots extracted from molecular dynamics (MD) simulations. The one-to-one mapping provided by the combination of simulation and experiment is in many cases powerful enough to allow the characterization of rather complex DNA aggregates [47]. This property chiefly stems from the atomic-scale resolution offered by MD, allowing the complex conformational space of large molecular aggregates to be sampled efficiently. Recently, we have applied this protocol to different G4 topologies, demonstrating its ability to discriminate between them, and even assisting in the identification of environment-driven phase transitions [48].

**Figure 1:**
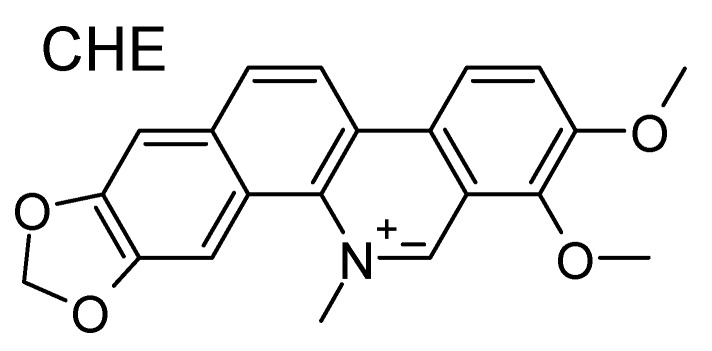
Molecular formula of chelerythrine (CHE). Note the positive charge center on the nitrogen atom.

Chelerythrine (CHE; Figure 1) is an organic ligand, the capacity of which to interact with DNA while experiencing a rather good selectivity for G4s has been recently established [50]. This benzophenantridine-based alkaloid possesses remarkable biological activity, including anticancer and antibacterial properties. CHE has been shown to be able to induce significant oxidative stress, both in vitro and in cell lines [51,52], while the CHE-mediated reactive oxygen species (ROS) increase has been correlated to its possible use as an anticancer agent. Notably, CHE’s high cytotoxic effects have been observed in human prostate cancer cell lines [53]. Interestingly enough, CHE has also been shown to possess antioxidant properties leading to neuroprotection [54]. Recently, the capacity of CHE to interact selectively with K^+^-stabilized G4 telomeric human DNA has been evidenced. In particular, a preference for parallel and hybrid G4s over antiparallel G4 has been put forth. The selective G4 binding capacity of CHE could constitute one of the reasons for its pharmacological activity.

In this contribution, we investigate the binding affinity of CHE toward B-DNA and different G4 conformations. Combination of molecular modeling and ECD allows the binding modes with DNA to be characterized precisely, while Ultra Violet-Visible (UV-Vis) titration provides the CHE–DNA binding constant (K_b_) and associated free energy (∆G) values. As a comparison, binding ∆G values are also determined by means of molecular mechanics simulations through enhanced sampling techniques based on alchemical transformations and free energy perturbation (FEP). Overall, the good agreement between calculated and experimental results obtained provides a comprehensive picture of the binding modes of CHE with both B-DNA and G4s in solution.

## 2. Materials and Methods

All chemicals, including chelerythrine, were purchased from Sigma Aldrich and used as received. Solvents were purchased as analytical grade and utilized without further purification. MilliQ water was used to prepare buffers and pH was measured using a Mettler Toledo pH meter. Analysis and plotting of the data were carried out using Origin 2017 (OriginLab Corporation, Northampton, MA, USA). All oligonucleotides were purchased from Integrated DNA Technologies (IDT) in high performance liquid chromatography (HPLC) purity grade. Here, 2HY9 corresponds to the human telomere sequence 5′-AAA GGG TTA GGG TTA GGG TTA GGG AA-3′ (26 bases); hTelo is also from human telomere but is shorter (22 bases): 5′- A GGG TTA GGG TTA GGG TTA GGG-3′; and *c-MYC* is the 27-base long oncogene promoter sequence: 5′- TGG GGA GGG TGG GGA GGG TGG GGA AGG-3′. The strand concentration of the oligonucleotide solutions was checked by measuring the absorbance at 260 nm and using the extinction coefficient values provided by the manufacturer. Lyophilized calf thymus DNA (ct-DNA, Sigma-Aldrich, Merck Group, Darmstadt, Germany) was utilized as a model for B-DNA. It was resuspended in 1.0 mM tris-hydroxymethyl-aminomethane (Tris-HCl) at pH 7.5. The DNA concentration (per base) was determined by UV spectrophotometry using 6600 M^−1^ cm^−1^ as the molar absorption coefficient at 260 nm.

### 2.1. Circular Dichroism and UV-Vis Spectroscopy

Thee circular dichroism spectra were recorded on Chirascan™ CD (by AppliedPhotophisics, Leatherhead, Surrey, UK), using 1 cm path-length quartz cuvettes at 25 °C. The UV-Vis spectra were collected on a PerkinElmer LAMBDA 35 double beam spectrophotometer, equipped with a Peltier temperature controller, and using 1 cm path-length quartz cuvettes. All experiments were carried out in 100 mM KCl, 50 mM Tris-HCl aqueous buffer, at pH 7.4. When necessary, we used NaCl instead of KCl. The lyophilized G4 oligonucleotides were dissolved in IDTE buffer (10 mM Tris, pH 7.5, 0.1 mM ethylenediamine tetraacetate (EDTA), Integrated DNA Technologies) to yield a 100 μM stock solution. This solution was then diluted using 50 mM Tris-HCl/100 mM KCl (or NaCl) buffer to the desired concentration. When indicated, we used polyethylene glycol (PEG) 200 as a crowding agent. The oligonucleotides were then folded into G4 by first heating the solutions up to 90 °C for 5 min and then by slowly cooling them down to room temperature. Calf thymus DNA (ct-DNA) stock solution was simply diluted using 50 mM Tris-HCl/100 mM KCl (or NaCl) buffer without any annealing procedure. The annealing of hTelo sequence in 100 mM NaCl buffered solution yielded a G4 in an antiparallel conformation, characterized by a negative ECD band around 260 nm and a positive band near 295 nm; the same sequence, annealed in 100 mM KCl buffered solution with 40% (*w*/*v*) of the crowding agent PEG 200, led to an all parallel structure, which showed a negative ECD peak near 240 nm and a positive peak near 265 nm. Finally, the hybrid (mixed parallel/antiparallel) structure, characterized by a negative peak near 240 nm and two positive peaks at 270 and 290 nm, was obtained by annealing 2HY9 in 100 mM KCl buffered solutions. ECD titrations (Figures S1–S5) were carried out by adding increasing amounts of CHE solution to a B-DNA/G4 solution with constant concentration. On the other hand, UV-Vis titrations (Figures S6–S10) were carried out by adding increasing amounts of B-DNA or G4 solution to a CHE solution with constant concentration. For comparison with the calculated ECD spectra, we selected the experimental curves where the ratio of CHE/DNA was ≥5, to ensure that the effect of CHE on the DNA structure was clearly visible. Full titrations are reported in ESI. K_b_ values were calculated by fitting the UV-Vis data at 316 nm (one of the bands of CHE where DNA does not absorb) through the binding model proposed by Thordarsonv [55]. The final equation was used as a script to fit the nonlinear curve of the software Origin 2017, as reported by Hargrove et al. [56]. For the fitting procedure, concentrations of the G4 oligonucleotides were converted to base concentration by multiplying the strand concentration by the number of bases in the sequence.

### 2.2. Modeling and Simulation

B-DNA was manually constructed using the Amber Nucleic Acid Builder (NAB) utility. In particular, we considered 14-base double strands of AT and CG sequences, respectively. More specifically, we constructed two extreme computational assays: homogenous DNA (ho-DNA), in which each strand was composed of one nucleotide type only (i.e., poly-d(A)-polyd(T) or poly-d(C)-polyd(G)), and heterogenous DNA (he-DNA), in which each strand contained the two alternating nucleotides (i.e., poly-d(AT)-polyd(TA) or poly-d(CG)-polyd(GC)). For G4, the structures of the parallel, antiparallel, and hybrid conformers were retrieved from the protein databank (pdb codes: 1KF1, 1XAV, 2Y9A, respectively). The interaction of CHE with both B-DNA and G4 was modeled by means of classical MD. To this end, DNA was represented by the Amber force field [57], including bsc0 corrections [58]. It is worth noting that run tests performed using the bsc1 [59] force field provided similar results. Water molecules were modeled with the TIP3P potential [60] and a minimal concentration of positive cations (K^+^) was added to the simulation box to ensure electric neutrality. The CHE force field was based on the generalized Amber force field (GAFF) [57], including a careful reparameterization of charges and some dihedrals (Figure S11). Equilibrium MD simulations were performed after manual construction of the DNA–CHE interacting complex, followed by minimization and thermalization. Equilibrium structures were sampled for 100 ns in the NPT ensemble (300K, 1 atm). All equilibrium MD trajectories were generated using the Graphics processing unit (GPU)-accelerated Amber 2015 code [61]. Representative and noncorrelated snapshots were extracted for each sample from the MD simulations in order to model the ECD properties. In this case, we resorted to a combination of QM/MM and a semi-empirical Frenkel Hamiltonian to describe the coupling between the chromophores [48,49,62]. All QM/MM calculations were performed using a locally modified version [63] of the Gaussian 09 D01 code [64] coupled with Tinker [65], and including the electronic response of the surroundings (ERS) to model polarizable embedding [66]. Details of the procedure, as well as the partition used for B-DNA and G4, are reported in Figure S12. However, for completeness, we remind the reader that this procedure consists of calculating the excitation energies and transition dipole moments of electronically excited states centered on each monomeric subunit at an ab initio level of theory. The final excitonic spectrum and the macromolecular ECD response are obtained by diagonalization of the Frenkel Hamiltonian, obtained as the scalar product of the transition dipole moments weighted by the relative distance between the subunits. Even though our model is extremely simple and more elaborate techniques based on an explicit use of the density matrix to describe the intermonomer coupling exist [67], it has proven efficient in reproducing the main features of nucleic acids in ECD.

### 2.3. Binding Free Energy Calculation

**Figure 2:**
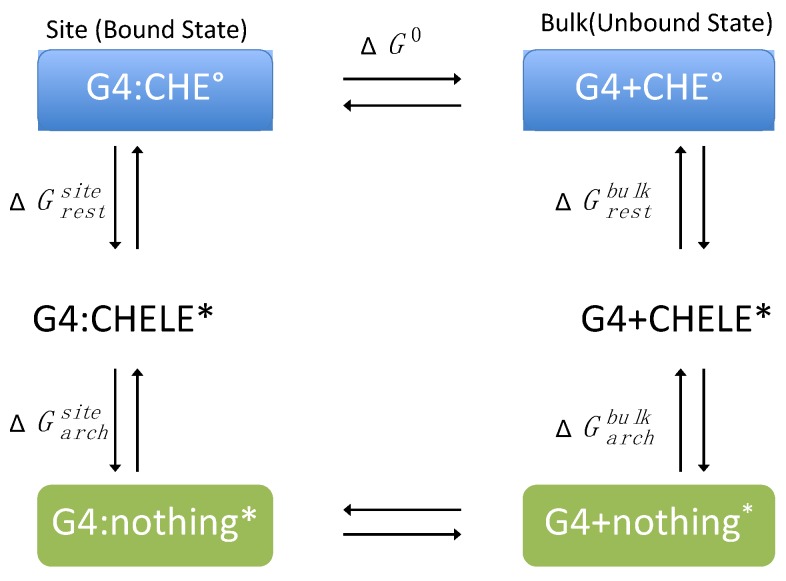
Thermodynamic cycle used for the calculation of the binding free energy (ΔG^0^) between CHE and the G-quadruplex (G4). The symbol * stands for a restrained state in the phase space.

The standard binding free energy, ΔG^0^, between CHE and DNA was evaluated following the alchemical cycle described in Figure 2 and Figure S13. In coupling and decoupling CHE from the hydrated G4/DNA environment or from the bulk, the position of CHE with respect to DNA was harmonically restrained by means of a set of spherical coordinates (r, θ, ϕ), whereas its relative orientation was restrained using the three Euler angles (Θ, Φ, Ψ) through the collective variable module (Colvars) [68] coupled with NAMD [69]. In this framework, the contribution caused by the restraints is evaluated rigorously and can be computed as:

(1) $$\Delta G^{0}=\Delta G_{rest}^{site}+\Delta G_{alch}^{site}+\Delta G_{rest}^{bulk}+\Delta G_{alch}^{bulk}$$

The free energy differences associated with the coupling–uncoupling of CHE from the hydrated double-strand DNA decamer and bulk water, represented by $\Delta G_{\mathrm{alch}}^{\mathrm{site}}$ and $\Delta G_{\mathrm{alch}}^{\mathrm{bulk}}$ , respectively, were computed within the FEP framework [70]. The reversible work, $\Delta G_{\mathrm{rest}}^{\mathrm{site}}$ and $\Delta G_{\mathrm{rest}}^{\mathrm{bulk}}$, associated with CHE positional or orientational restraints and with DNA conformational restraint, respectively, were evaluated using thermodynamic integration (TI) simulations or analytically [71] (see Table S1 in Supplementary Information for more details).

## 3. Results

### 3.1. Interaction with B-DNA

**Figure 3:**
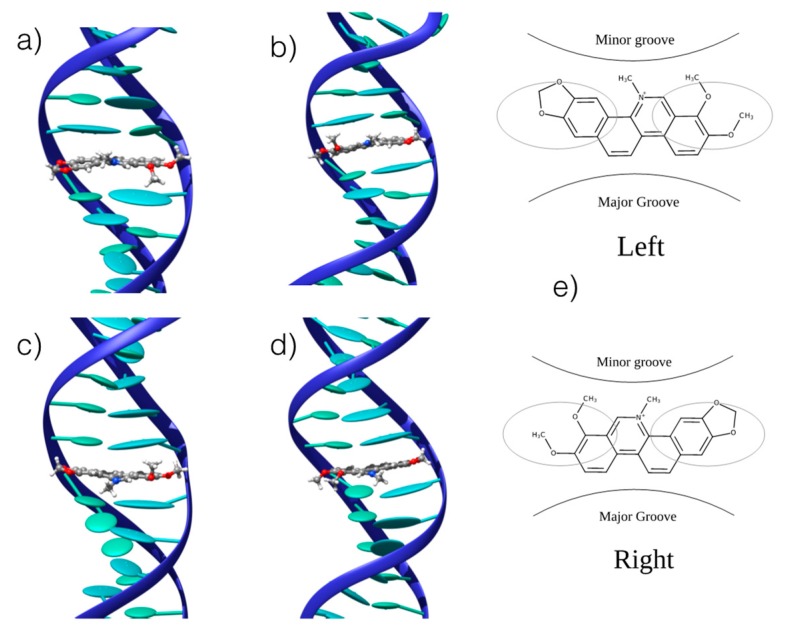
Representative snapshots of the stable interaction modes between CHE and B-DNA: (**a**) he-AT left orientation; (**b**) he-AT right orientation; (**c**) ho-AT left orientation; (**d**) ho-AT right orientation. (**e**) Schematic representation of the of the two competitive left and right interaction modes. The ellipsoids represent the π-stacked nucleobases. CG strands give similar structures that are reported in the Supplementary Information.

We confirmed the existence of stable interaction modes between CHE and B-DNA by performing UV-Vis absorption spectroscopic titration (see Supplementary Information), observing a change in the absorption spectrum as a function of the CHE concentration. The UV-Vis absorption titration was further used to determine the binding free energies, as reported in the following section. MD was conducted using double-helix DNA. We considered two different arrangements: homologous (ho-DNA) and heterogeneous (he-DNA) self-complementary sequences (see Methodology for additional detail). The simulations revealed the formation of stable adducts, both in the case of poly-(dAdT) and poly-(dGdC) oligomers. More specifically, we consistently observed a persistent intercalation mode for ho-DNA and he-DNA. However, two different positions of CHE can be observed, corresponding to a mirror left/right arrangement of the CHE cyclic acetal moiety with respect to the DNA axis, leading to a total of four different binding modes with B-DNA (Figure 3).

**Figure 4:**
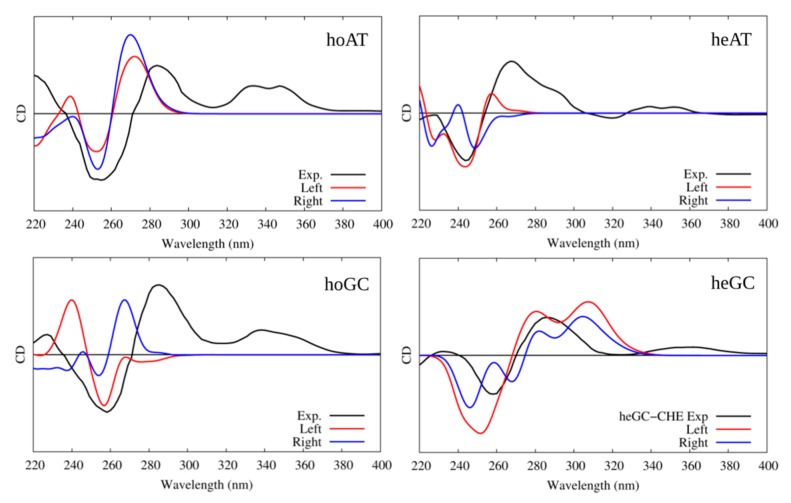
Experimental and simulated electronic circular dichroism (ECD) spectra of CHE in the presence of B-DNA; intensities are reported in arbitrary units. The black line represents the experimental spectrum uniformly scaled to match the values of the convoluted one. The red line represents the simulated spectrum for CHE in the left orientation, while the blue one represents the simulated ECD of CHE in the right orientation. Note that the CHE absorption contribution was not considered in the calculated spectra.

In all cases, the B-DNA–CHE adducts appear stable and persistent all along the MD simulations, while the structural deformations induced on the DNA helix are mostly reminiscent of typical intercalation modes, most notably presenting an increase of the interbase distance in correspondence with the intercalation pocket. The global helical bending, on the other hand, does not exhibit significant deviations from the ideal B-DNA values. The comparison between the experimental and the simulated ECD spectra is reported in Figure 4. It must be noted that our QM partition was specifically designed to consider only the contribution arising from the DNA nucleobases, hence, no CHE absorption band is present. This choice allows us to focus on the structural deformations induced by the ligand as reflected in the ECD spectra.

One may observe that the global shape of the experimental ECD spectra is well reproduced by the simulated results once a proper energy shift is applied. Note that the energy shift is necessary due to the use of a very simplified phenomenological Hamiltonian, such as the Frenkel coupling, and is also system-dependent and can reach values close to the eV. However, in this contest, and to allow structural observations to be rationalized, the correct reproduction of the band shape is much more important than the recovery of the absorption energies. In detail, we reproduced the typical signature characterized by the presence of a positive band at around 275–280 nm followed by a negative one at about 245–250 nm. Interestingly, in all cases, the spectra for left and right orientation of CHE are different, however, this comparison alone is not sufficient to definitively assign the dominant orientation. This aspect is particularly striking for CG strands, for which the differences in the ECD profiles are much more obvious.

### 3.2. Interaction with G4 DNA 

**Figure 5:**
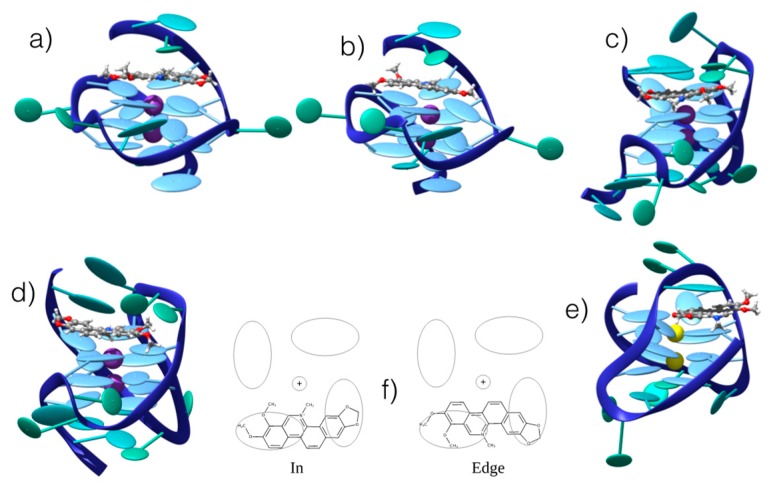
Representative snapshots of the persistent interaction modes between CHE and G4 in hybrid, parallel, and antiparallel conformations from different sequences (see Methods): (**a**) “edge”/parallel; (**b**) “in”/parallel; (**c**) “edge”/hybrid; (**d**) “in”/hybrid; (**e**) “edge”/antiparallel. (**f**) Schematic representation of the competitive “in” and “edge” interaction modes. The ellipsoids represent π-stacked nucleobases in the G4 core. Note that for antiparallel G4, only one stable interaction mode has been obtained.

As far as G4 is concerned, the MD simulations confirmed the presence of stable and persistent aggregates with CHE (Figure 5). All the G4 conformations (parallel, hybrid, and antiparallel) showed, indeed, the capacity to interact with CHE. In all cases, the interaction appears to occur through top or end-stacking, with CHE residing on top of the tetrad of the G4. Such a disposition is mostly driven by π-stacking and dispersion interactions of the extended conjugated system of CHE and the four guanine bases, as well as by electrostatic interaction of the positively charged aromatic ligand and the negatively charged sugar-phosphate DNA backbone. However, as in the case of B-DNA, two different interaction modes can be identified: “in” and “edge”, the two differing by the relative orientation of the CHE nitrogen atom with respect to the G4 center. In fact, in the “in” spatial arrangement, the positive nitrogen points towards the internal G4 channel, while in the “edge” arrangement, it is oriented towards the tetrad periphery and the DNA backbone (Figure 5). Although all the G4s lead to stable aggregates, some differences can be noticed. Most importantly, while parallel and hybrid arrangements give rise to both “in” and “edge” conformations, the antiparallel G4 only adopts the “edge” mode. Indeed, MD simulations starting from the “in” conformation invariably resulted in either the disruption of the interaction or the evolution towards the “edge” arrangement. This preference can be explained both by the different cations filling the central channel for this particular G4 (i.e., Na^+^ versus K^+^), and by the fact that the backbone arrangements provide different interaction pathways between the positively charged nitrogen of CHE and the negatively charged phosphate group of DNA. Such electrostatic interaction is, in fact, less pronounced for the other conformers. In this respect, the role of the ionic strength and the nature of the ions in modulating the structure and dynamics of DNA either in canonical or non-canonical arrangement goes beyond the scope of the present contribution, and would require an entire study. As a consequence, we consistently constrain ourselves to the use of a minimum salt concentration environment (meaning we only provide the cation needed to assure electroneutrality).

**Figure 6:**
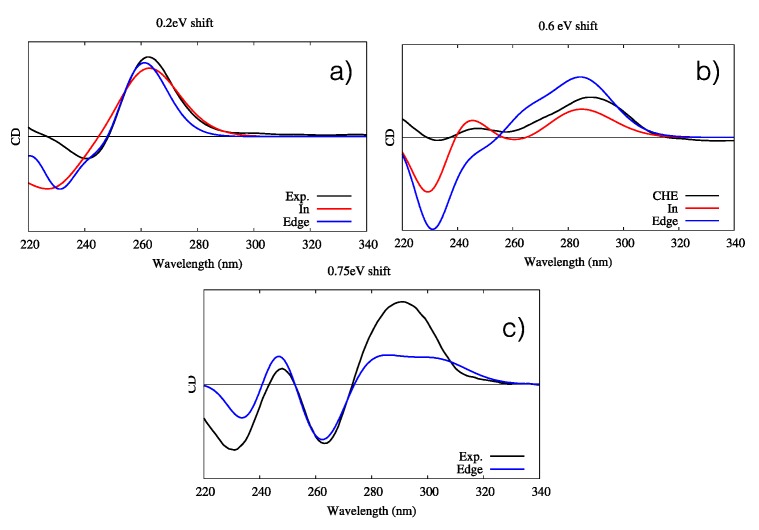
Experimental and simulated ECD spectra of the three G4–CHE complexes. Intensities are given in arbitrary units. (**a**) Parallel conformation, including the experimental (black line) and simulated “in” (red line) and “edge” (blue line) orientations; a shift of 0.2 eV has been applied. (**b**) Hybrid conformation, including the experimental (black line) and simulated “in” (red line) and “edge” (blue line) orientations; a shift of 0.6 eV has been applied. (**c**) Antiparallel conformation, including the experimental (black line) and simulated “edge” orientations (blue line); a shift of 0.75 eV has been applied. Note that for the simulated spectra, all stable interaction modes for each G4 conformer have been considered. The quantum mechanics (QM) partition was specifically designed to consider the DNA contribution only; hence, the CHE signal is not considered in the simulated spectra.

The interaction of CHE with the selected G4s was, once again, confirmed by UV-Vis absorption spectroscopy (see Supplementary Information) and by ECD, as reported in Figure 6. Just as before, the experimental ECD were compared with the simulated spectra obtained by sampling the stable conformation of each G4 topology, following a protocol reported in a previous work [48]. Compared to B-DNA, the resolution of the CHE interaction mode on the base of the sole ECD is more complex because of the higher similarity of the ECD spectra for all studied cases. This fact is not surprising because of the much higher rigidity of the G4 core compared to B-DNA, and hence, its much smaller structural deformation due to the binding with CHE. All G4 topologies yield an ECD spectrum dominated by a large positive band at around 280 nm. Additionally, while the antiparallel arrangement features a symmetric negative–positive–negative ECD motif peaking at 260, 240, and 230 nm, the other G4 topologies possess a far less complex structure. For these, the parallel arrangement shows, in addition to the positive peak at longer wavelengths, a weak negative band at 240 nm, while the hybrid one gives rise to two very weak positive and negative additional peaks at 240 and 230 nm, respectively.

The agreement between the simulated and experimental spectra is good. Once again, this is likely by virtue of the enhanced rigidity of G4s, in particular as far as the band shapes are concerned. However, an energy shift is needed because of the use of a very simple Frenkel Hamiltonian. Indeed, the use of more elaborated methodologies, including the coupling of the transition density matrices instead of the transition dipole moment, could allow reduction of the energy shift. In the case of the parallel arrangement, both “in” and “edge” conformations give almost undistinguishable signals, making their identification virtually impossible. In the case of the antiparallel arrangement, while both conformations correctly reproduce the positive and bright peak at 260 nm, only the “in” conformation is able to yield the correct positive–negative–positive motif of the spectrum, even though the intensity ratio is less well-reproduced. Such results clearly point out the preference of CHE for the “in” binding mode. Finally, all features of the antiparallel G4’s ECD spectra are correctly reproduced by the “edge” conformation, even though the intensity of the dominant positive peak at lowest energies is underestimated compared to that of the experimental spectrum. Globally, our results prove the capacity of our protocol to correctly reproduce the main features of ECD spectra of complex systems, while also helping to unveil the complex scenario whereby the interaction with external drugs, in particular their relative conformations and modes of binding, is strongly dependent on the G4 topology. For instance, while the antiparallel arrangement shows a marked preference for the “edge” mode of binding, the opposite is true for the hybrid G4.

### 3.3. Determination of Binding Constants and Free Energies

In order to quantitatively characterize the binding of CHE with the selected DNA sequences and to clarify the selectivity towards G4 versus B-DNA on the one hand, and the preferential affinity for different quadruplex topologies on the other hand, we determined the CHE–DNA binding constant, K_b_, by spectrophotometric UV-Visible absorption titrations (see Material and Methods section and the spectra in the Supplementary Information). The obtained results, also expressed in terms of binding free energies (ΔG), are reported in Table 1. As first evidence, the preferential binding affinity towards G4 is confirmed by the K_b_ values. Indeed, the binding strength towards B-DNA is at least one order of magnitude lower than that of all G4 topologies, regardless of the central ions used or the presence of crowding agents. Conversely, no clear selectivity towards one specific G4 sequence or arrangement clearly arises, likely because of the fact that CHE acts as a top binder. Indeed, all the G4 sequences have binding constants on the order of 10^−5^ M^−1^, with just a small preference for the human telomeric sequence. The inclusion of the crowding agent, triggering the transition from parallel to antiparallel, seems to increase the affinity. However, one ought to consider that precise disentanglement of the effects of the crowding agent itself and the conformational transition are not straightforward. Even though the selectivity towards specific G4 arrangements is far from being optimal, the slight preference of CHE towards binding with telomere sequences, irrespective of their arrangements, is promising for the design of new and more specific telomerase inhibitors via G4 targeting. The observed global trend is also confirmed by the calculation of binding free energies for two specific G4 conformations, namely, hybrid and parallel, the values of which are between −12 and −13 kcal/mol. Consistent with the experimental results, no clear preference for one conformer is observed, since the differences between the two binding free energies fall within the error bars of these calculations. The slight deviation from the experimental absolute values, and in particular the overestimation of the DNA binding free energies, may be ascribed to two factors. First, our simulations were carried out at minimal salt concentration compared to the experimental conditions. Higher ionic strength is known to decrease the electrostatic interaction of the positively charged CHE and the DNA backbone because of the screening of the electrostatic potential. Second, the force field for G4 is far less-developed than for that double helical DNA, and these potential energy functions are often not tested and parameterized to reproduce thermodynamic properties such as binding constants.

## 4. Conclusions

The binding mode and strength of the alkaloid CHE interaction with DNA strongly depends on the DNA sequence and topology. In particular, the title molecule possesses a preferential affinity for G-quadruplexes versus B-DNA of at least one order of magnitude. MD simulations followed by QM/MM calculations readily provide DNA binding free energies and simulated ECD spectra in excellent agreement with the available experimental data, thereby convincingly complementing the structural models inferred from spectroscopic determinations. In particular, it has been possible to point to significant differences in the simulated spectra of the left and right intercalation modes with B-DNA, and between edge and in stacking modes with parallel, hybrid, and antiparallel G-quadruplexes. The results obtained in the present work underscore that our proposed multiscale modeling protocol provides support for the interpretation of the drug–target interaction, and as a consequence, for a computationally guided drug design. Indeed, this protocol allows for the elucidation and prediction of the binding mechanism between small drug candidates and biomolecular targets, overcoming the intrinsic difficulties of expensive techniques such as X-ray crystallography or high-resolution nuclear magnetic resonance (NMR). Furthermore, this combined experimental computational method based on CD is envisioned to be extremely helpful in the case of the analysis of multiple G4s (e.g., in case of the gene promoter), which contains three adjacent quadruplexes [72]), where neither crystallography nor NMR structures are available to date.

## Figures and Tables

**Table 1:** Experimental binding constants (K_b_) and binding free energies (ΔG) for the CHE–DNA complexes. The calculated ΔG values are also shown for comparison.

DNA	Experimental K_b_ (M^−1^)	Experimental ΔG (kcal/mol)	Calculated ΔG (kcal/mol)
B-DNA	(1.3 ± 0.2) × 10^4^	−5.6 ± 0.1	
Hybrid G4 (2Y9A)	(1.1 ± 0.1) × 10^5^	−6.9 ± 0.1	−12.3
Parallel G4 (*c-MYC*)	(1.0 ± 0.1) × 10^5^	−6.8 ± 0.02	−13.5
Antiparallel G4 (hTelo)	(2.69 ± 0.06) × 10^5^	−7.4 ± 0.2	
Parallel G4 (hTelo + PEG)	(3.5 ± 0.9) × 10^5^	−7.6 ± 0.1	

## Supplementary Materials

The following are available online at https://www.mdpi.com/2076-3921/8/10/472/s1. Figure S1: CD titration of 2H19 G4 with increasing aliquots of CHE. Below, the corresponding UV-Vis spectra. Buffer: 50 mM Tris-HCl and 100 mM KCl, pH 7.4. Figure S2: CD titration of hTelo (Na^+^) G4 with increasing aliquots of CHE. Below, the corresponding UV-Vis spectra. Buffer: 50 mM Tris-HCl and 100 mM NaCl, pH 7.4. Figure S3: CD titration of c-MYC G4 with increasing aliquots of CHE. Below, the corresponding UV-Vis spectra. Buffer: 50 mM Tris-HCl and 100 mM KCl, pH 7.4. Figure S4: CD titration of hTelo G4, in the presence of 40% (w/v) PEG 200, with increasing aliquots of CHE. Below, the corresponding UV-Vis spectra. Buffer: 50 mM Tris-HCl and 100 mM KCl, pH 7.4. Figure S5: CD and corresponding UV-Vis spectra of CHE. Buffer: 50 mM Tris-HCl and 100 mM KCl, pH 7.4. CHE is not chiral, and accordingly, does not induce any CD signal. UV-Vis titration of CHE with B-DNA. In the inset is the plot of the data (at 316 nm) with fitting curve (in red) for the determination of the Kb value. Buffer: 50 mM Tris-HCl and 100 mM KCl, pH 7.4. Figure S7: UV-Vis titration of CHE with 2HY9 G4. In the inset is the plot of the data (at 316 nm) with fitting curve (in red) for the determination of the Kb value. Buffer: 50 mM Tris-HCl and 100 mM KCl, pH 7.4. Figure S8: UV-Vis titration of CHE with hTelo (Na+) G4. In the inset is the plot of the data (at 316 nm) with fitting curve (in red) for the determination of the Kb value. Buffer: 50 mM Tris-HCl and 100 mM NaCl, pH 7.4. Figure S9: UV-Vis titration of CHE with c-MYC G4. In the inset is the plot of the data (at 316 nm) with fitting curve (in red) for the determination of the Kb value. Buffer: 50 mM Tris-HCl and 100 mM KCl, pH 7.4. Figure S10: UV-Vis titration of CHE with hTelo G4 in the presence of 40% (*w*/*v*) PEG 200. In the inset is the plot of the data (at 316 nm) with fitting curve (in red) for the determination of the Kb value. Buffer: 50 mM Tris-HCl and 100 mM KCl, pH 7.4. Figure S11: Molecular formula of CHE, indicating the modified dihedral angle. Figure S12: Partitioning of the macromolecular aggregate used to obtain the semiempirical Frenkel Hamiltonian in the case of B-DNA (left) and G4 (right). The red squares represent the chunks included in the QM partition. Note that in all cases, three stacked nucleobases are simultaneously included in each partition, hence allowing for the excited states to be delocalized over the whole subsystem. Figure S13: Thermodynamic cycle using the calculation of the binding free energy. The free energy terms are indicated. Table S1: Calculated free energy components for the binding of CHELE to G4 DNA.
