# Supplementary material for: Targeting G-quadruplexes with Organic Dyes: Chelerythrine–DNA Binding Elucidated by Combining Molecular Modeling and Optical Spectroscopy
Source: Antioxidants (Basel). 2019 Oct 10;8(10):472. doi: 10.3390/antiox8100472 (PMC6826623; doi:10.3390/antiox8100472)
Supplement: Supplementary file 1 [file antioxidants-08-00472-s001.pdf]

**Supplementary Information for:**

# Targeting G-quadruplexes with organic dyes: Chelerythrine-DNA binding elucidated combining molecular modeling and optical spectroscopy.

**Alessio Terenzi,[1,2,*] Hugo Gattuso,[3] Angelo Spinello,[4] Bernhard K. Keppler,[1] Cristophe Chipot,[3,5,6] François Dehez,[3,5] Giampaolo Barone,[7,*] and Antonio Monari.[3,*]**

[1]Institute of Inorganic Chemistry, University of Vienna, Währingerstrasse 42, Vienna, Austria.
[2]Donostia International Pjhysics Center, Paseo Manuel de Lardizabal 4, Donostia 20018, Spain.
[3]Université de Lorraine and CNRS, LPCT UMR 7019, F54000 Nancy, France.
[4]CNR-IOM DEMOCRITOS c/o International School for Advanced Studies (SISSA), Trieste, Italy.
[5]Laboratoire International Associé Centre National de la Recherche Scientifique et University of Illinois at Urbana−Champaign.
[6]Department of Physics, University of Illinois at Urbana−Champaign, 1110 West Green Street, Urbana, Illinois 61801, United States.
[7]Dipartimento di Scienze Biologiche, Chimiche e Farmaceutiche, Università di Palermo, Viale delle Scienze, Palermo, Italy.

* Correspondence: aterenzi@dipc.org, giampaolo.barone@unipa.it, antonio.monari@univ-lorraine.fr.

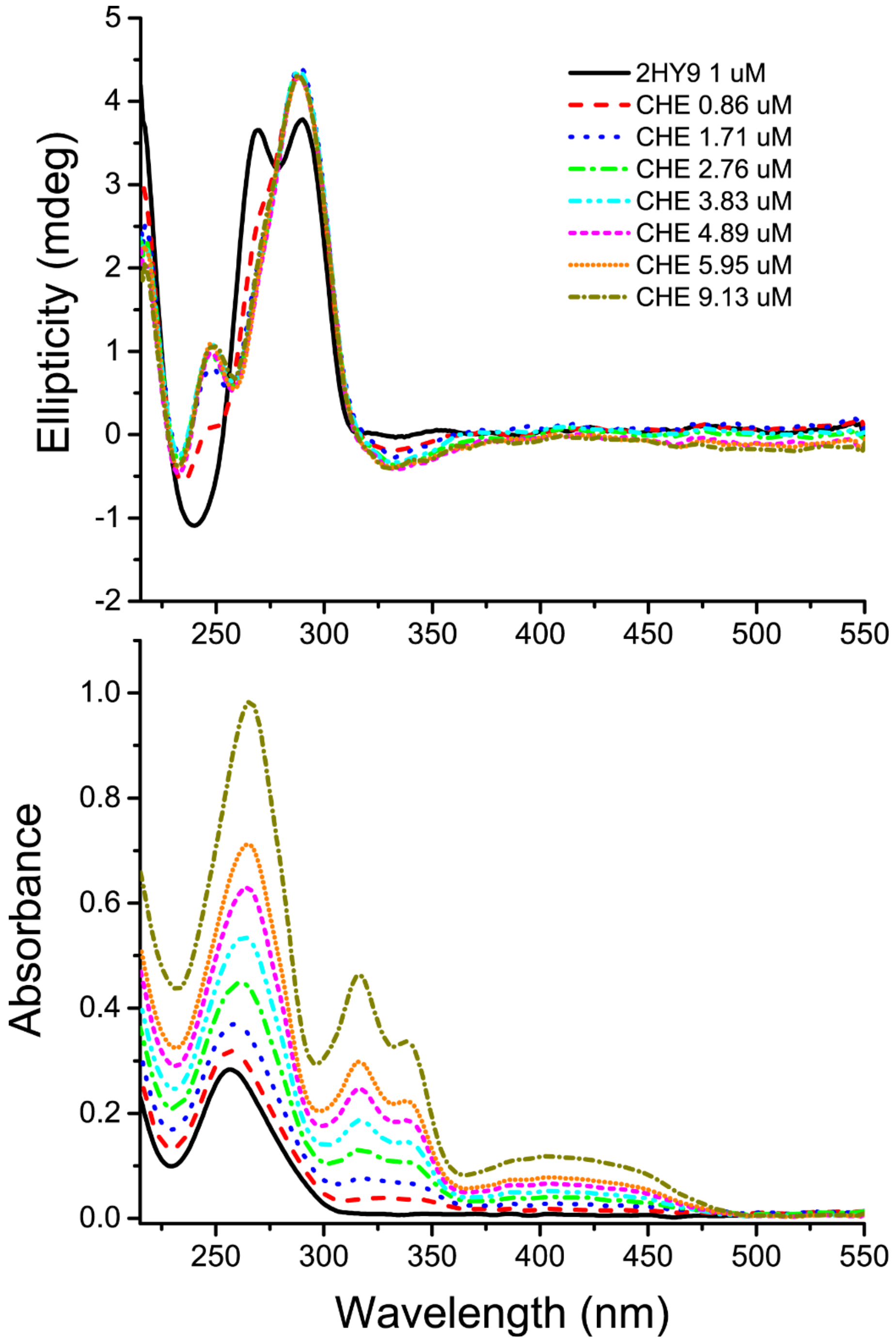


**Figure S1.** CD titration of 2H19 G4 with increasing aliquots of CHE. Below, the corresponding UV-Vis spectra. Buffer: 50 mM Tris-HCl and 100 mM KCl, pH 7.4.

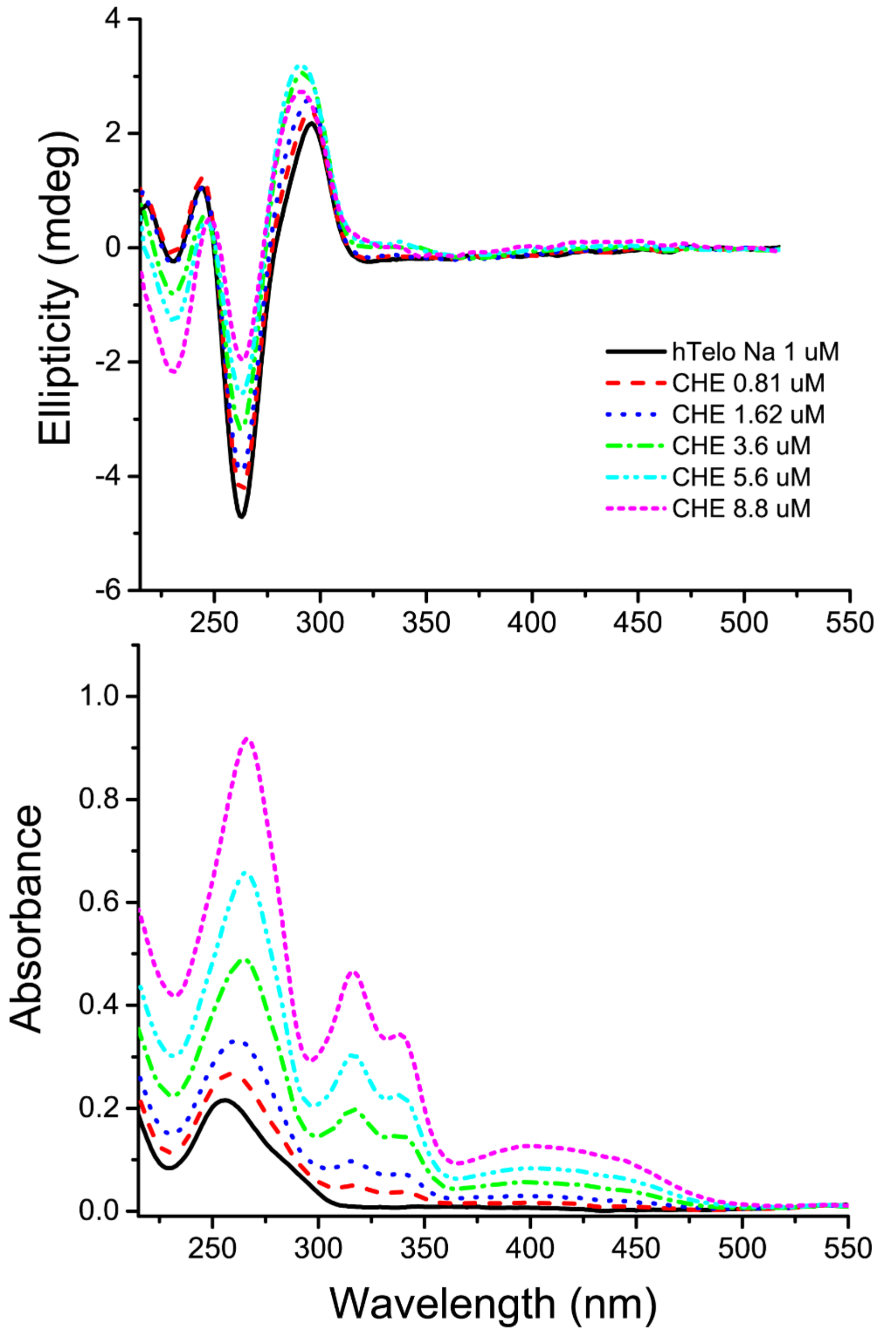


**Figure S2.** CD titration of hTelo ($Na^+$) G4 with increasing aliquots of CHE. Below, the corresponding UV-Vis spectra. Buffer: 50 mM Tris-HCl and 100 mM NaCl, pH 7.4.

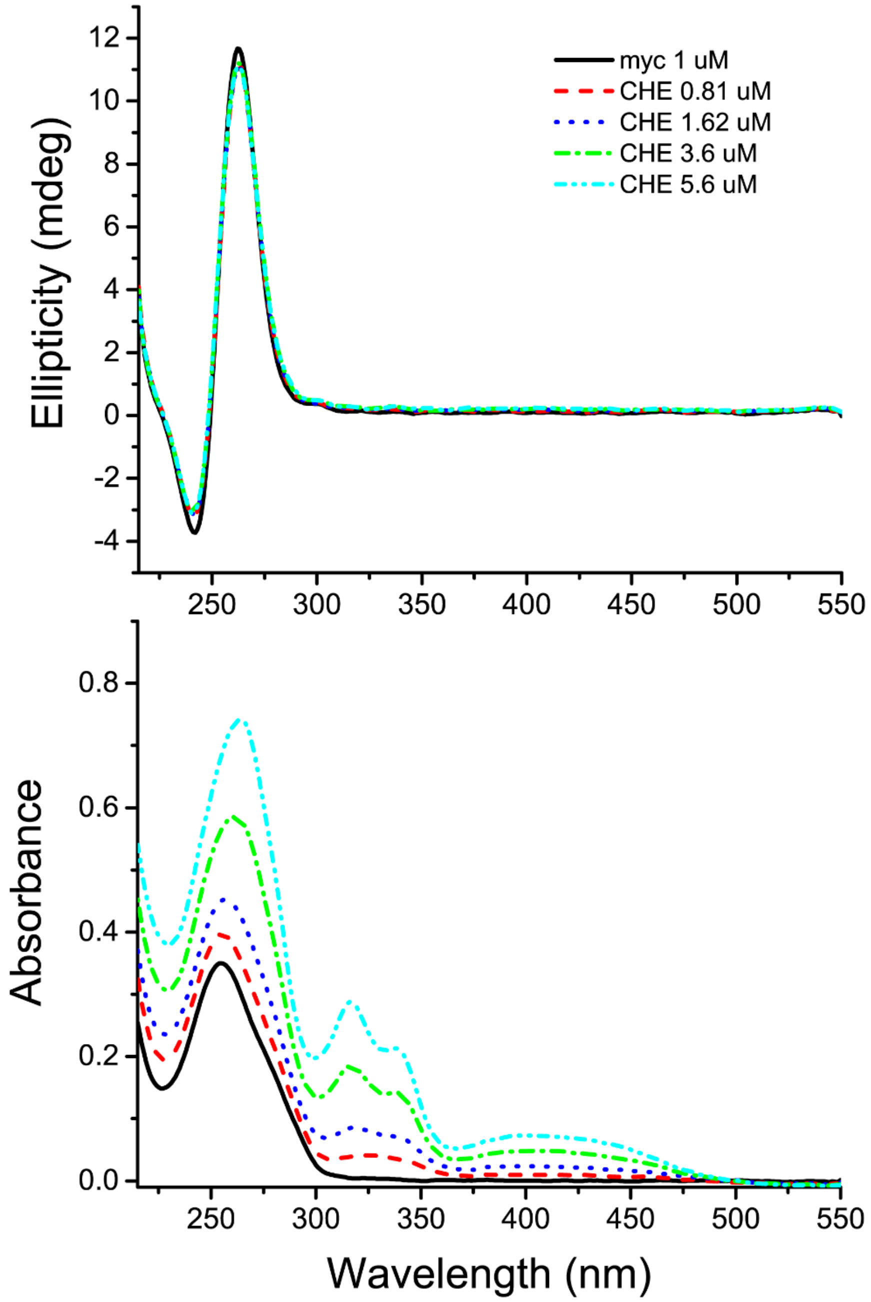


**Figure S3.** CD titration of *c-MYC* G4 with increasing aliquots of CHE. Below, the corresponding UV-Vis spectra. Buffer: 50 mM Tris-HCl and 100 mM KCl, pH 7.4.

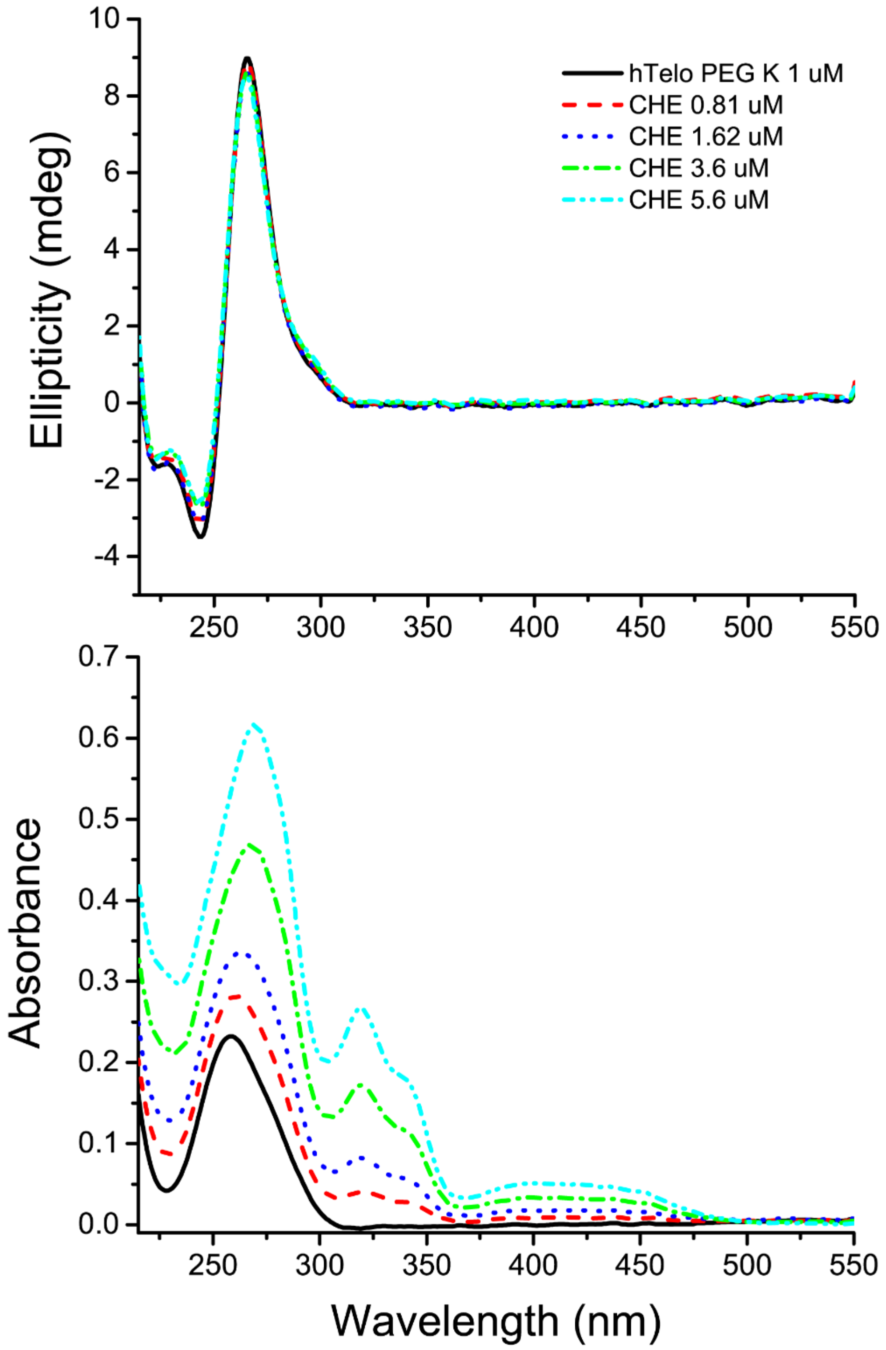


**Figure S4.** CD titration of hTelo G4, in the presence of 40% (w/v) PEG 200, with increasing aliquots of CHE. Below, the corresponding UV-Vis spectra. Buffer: 50 mM Tris-HCl and 100 mM KCl, pH 7.4.

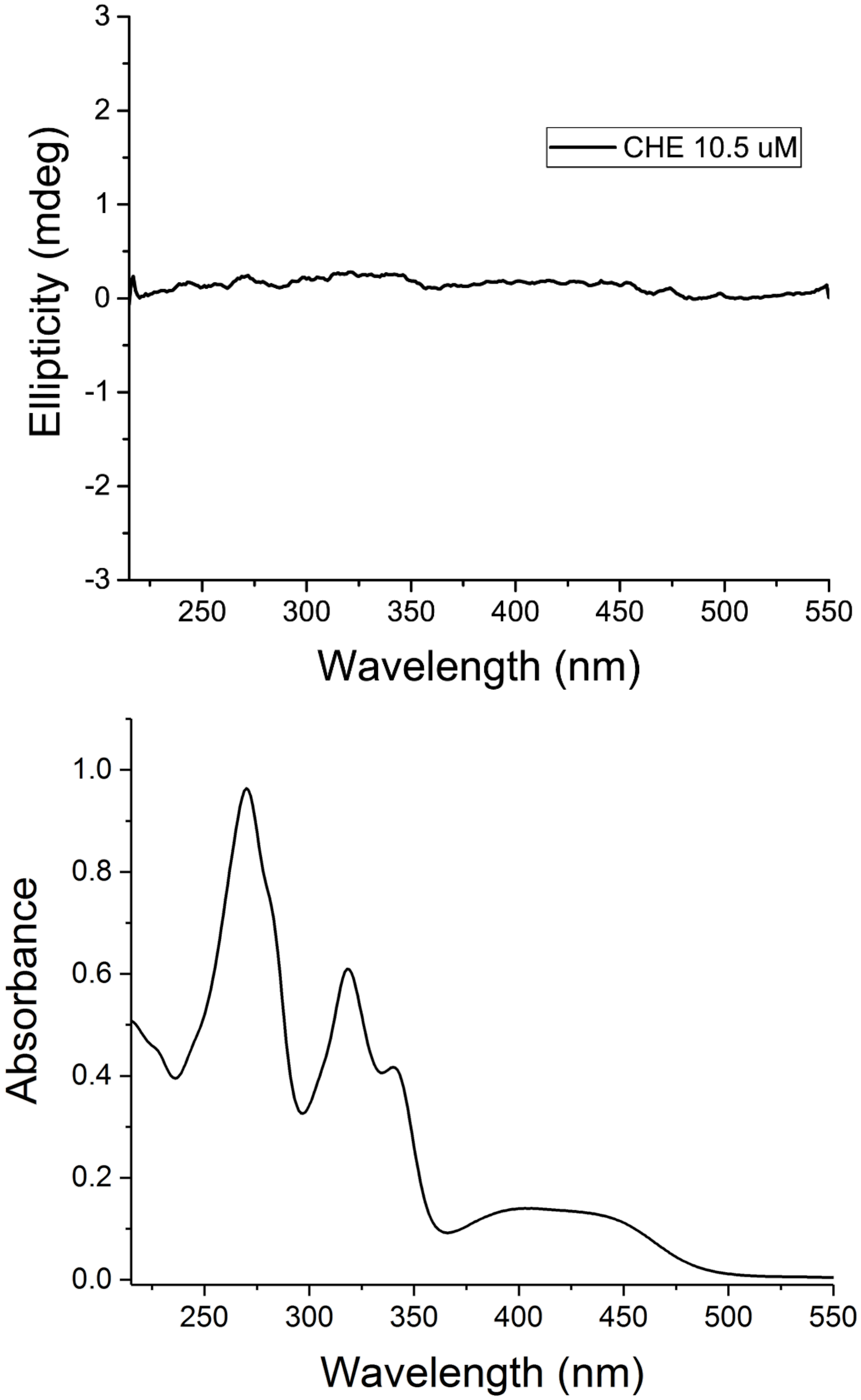


**Figure S5.** CD and corresponding UV-Vis spectra of CHE. Buffer: 50 mM Tris-HCl and 100 mM KCl, pH 7.4. CHE is not chiral and, accordingly, does not induce any CD signal.

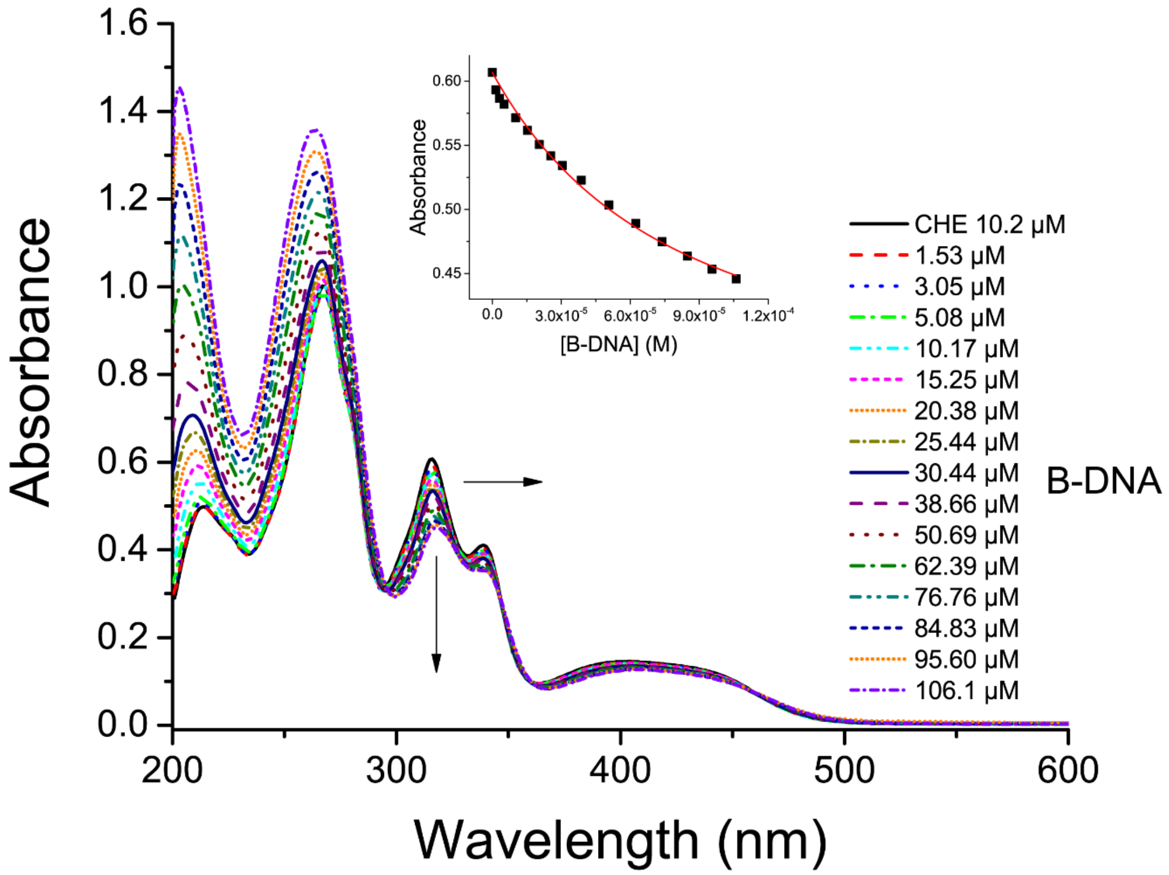


**Figure S6.** UV-Vis titration of CHE with B-DNA. In the inset the plot of the data (at 316 nm) with fitting curve (in red) for the determination of the $K_b$ value. Buffer: 50 mM Tris-HCl and 100 mM KCl, pH 7.4.

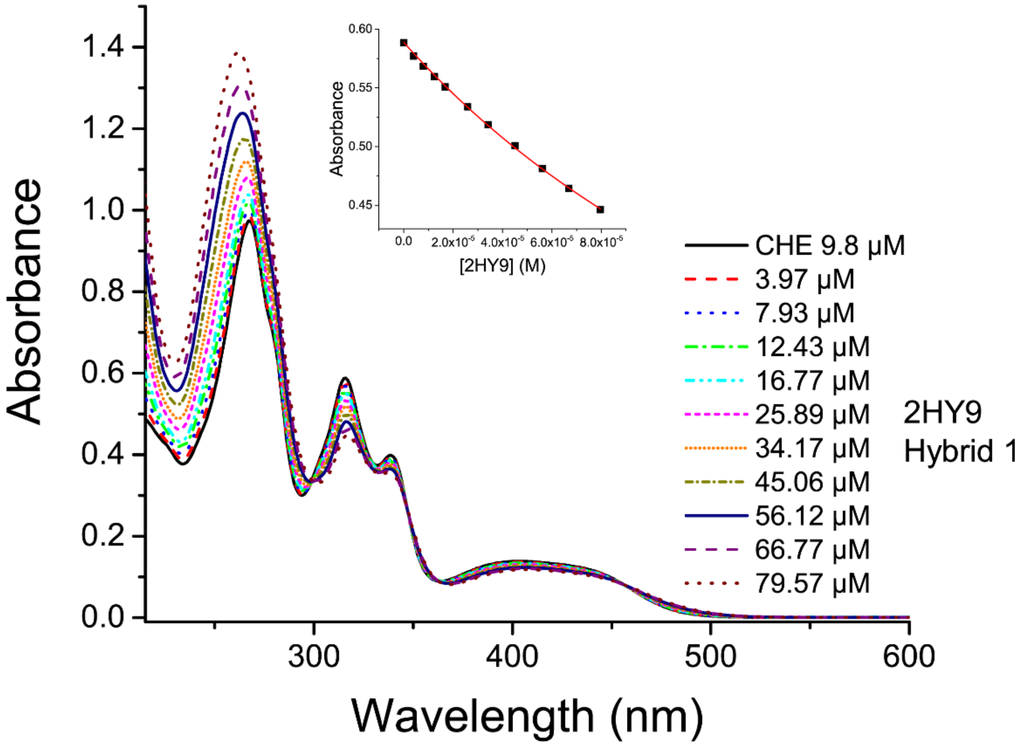


**Figure S7.** UV-Vis titration of CHE with 2HY9 G4. In the inset the plot of the data (at 316 nm) with fitting curve (in red) for the determination of the $K_b$ value. Buffer: 50 mM Tris-HCl and 100 mM KCl, pH 7.4.

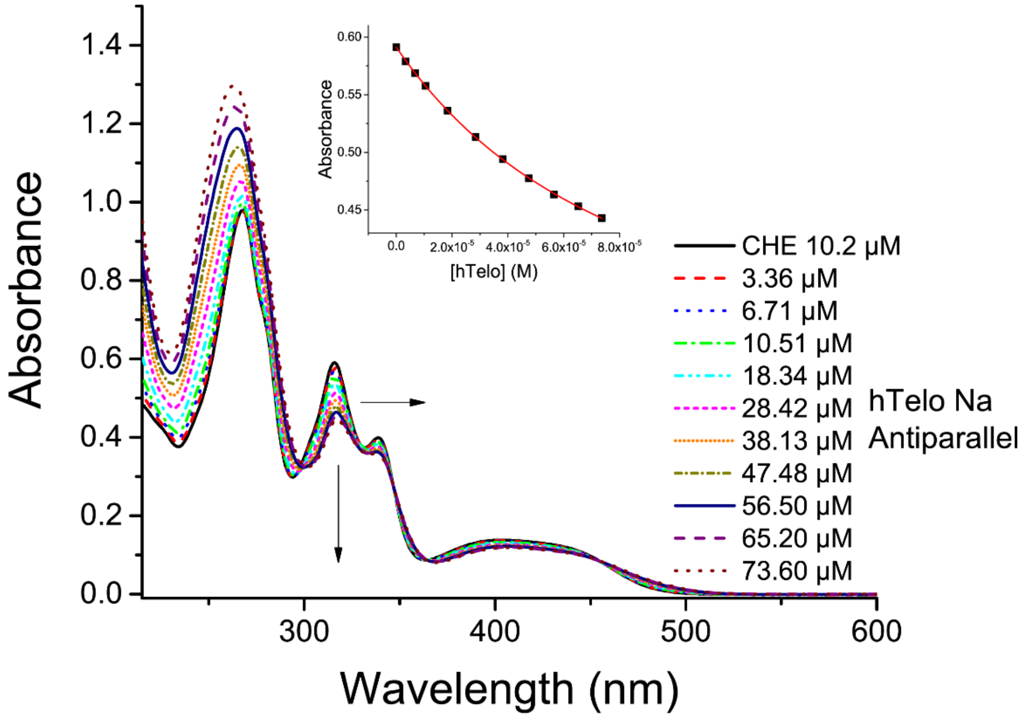


**Figure S8.** UV-Vis titration of CHE with hTelo ($Na^+$) G4. In the inset the plot of the data (at 316 nm) with fitting curve (in red) for the determination of the $K_b$ value. Buffer: 50 mM Tris-HCl and 100 mM NaCl, pH 7.4.

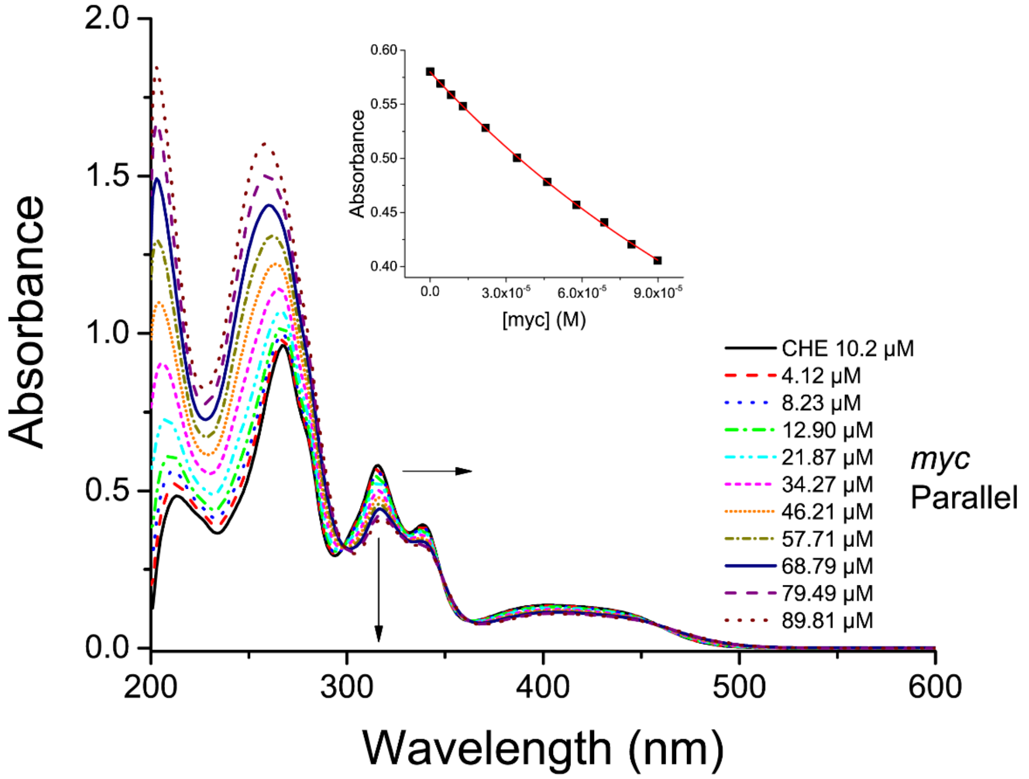


**Figure S9.** UV-Vis titration of CHE with *c-MYC* G4. In the inset the plot of the data (at 316 nm) with fitting curve (in red) for the determination of the $K_b$ value. Buffer: 50 mM Tris-HCl and 100 mM KCl, pH 7.4.

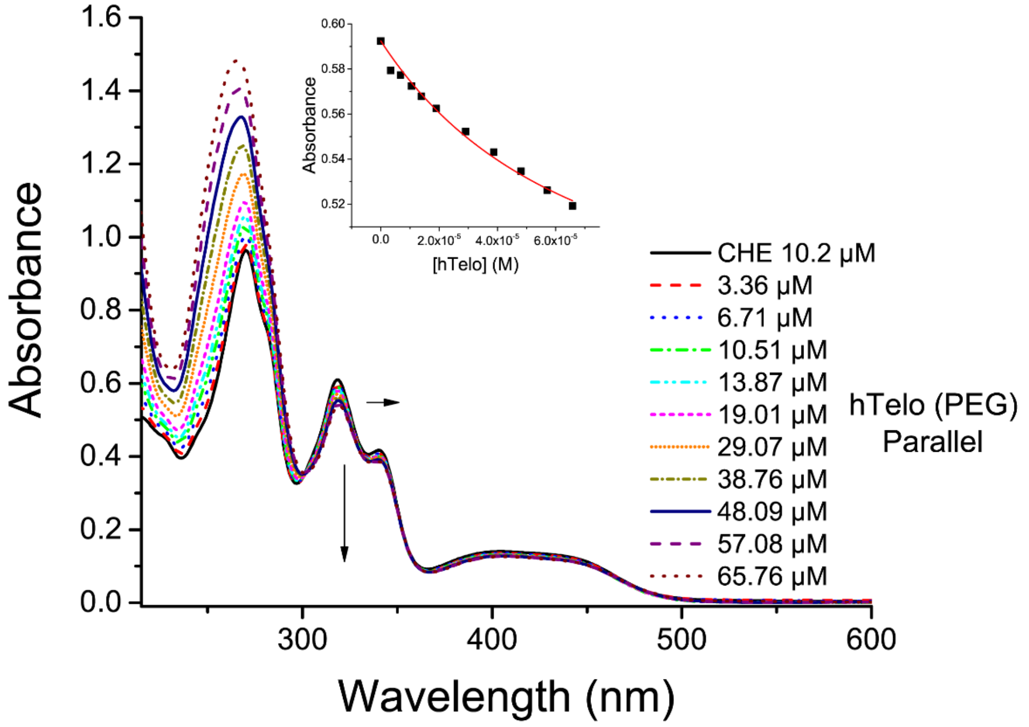


**Figure S10.** UV-Vis titration of CHE with hTelo G4 in the presence of 40% (w/v) PEG 200. In the inset the plot of the data (at 316 nm) with fitting curve (in red) for the determination of the $K_b$ value. Buffer: 50 mM Tris-HCl and 100 mM KCl, pH 7.4.

## Details on Molecular Modeling and Simulations

*Chelerythrine:*

The equilibrium ground state geometry of the chelerythrine molecule has been optimized at the DFT B3LYP/6-31G(d) level of theory. Atomic point charges have been obtained through the RESP procedure REF using HF/6-31G*. Both computations have been performed using Gaussian D.01. Then the bonded and non-bonded parameters for molecular mechanics and molecular dynamics simulations are GAFF parameters REF adapted to chelerythrine using the antechamber utility available in Ambertools. It has to be noted that the force constant describing the dihedral between atoms 1, 2, 3 and 4 as depicted in Figure S11 has been increased (from 0.300 to 3.625 kcal/mol) to obtain an identical population of conformations where the methyl is pointing out of the plane of the molecule either on one side or the other.

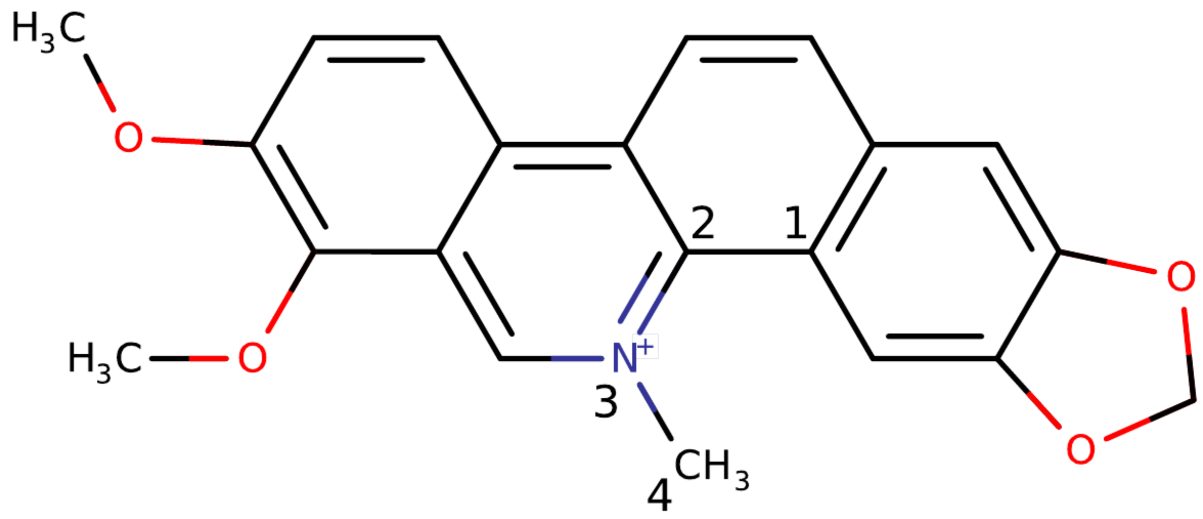


**Figure S11.** Molecular formula of CHE, indicating the modified dihedral angle.

*Equilibrium Molecular dynamics simulations:*

Starting structures of the 14 base pairs long B-DNA double strands heteropolymers of AT and GC have been obtained using the NAB utility available in Ambertools. In the case of G-quadruplexes, parallel, hybrid and antiparallel configurations are from the ProteinDataBank, respectively 1XAV, 2HY9 and 143D. For all the complexes described in the Introduction section, the starting conformations has been created manually using the Avogadro software. Then each system has been placed in a truncated octahedron box of water molecules described in subsequent molecular mechanics and molecular dynamics simulations by the TIP3P parameters. Amber99 force field including bsc1 corrections has been used to describe nucleic acids. In the case of B-DNA/chelerythrine the systems contained approximatively 7650 water molecules and 25 $K^+$ counter-ions to neutralize the global charge of the system. For G4/chelerythrine the systems contained: for parallel 4860 water molecules and 18 $K^+$ ions (plus the two central $K^+$ ions inside the G4 central channel), for hybrid 4800 water molecules and 18 $K^+$ ions (plus the two central $K^+$ ions within the G4) , for antiparallel 4630 water molecules and 18 $K^+$ ions (plus the two central $Na^+$ ions within the G4).

The molecular mechanics followed by molecular dynamics simulations procedure we employed was identical for the 10 studied systems (poly(A·T)·poly(A·T)/CHE left, poly(A·T)·poly(A·T)/CHE right, poly(G·C)·poly(G·C)/CHE left, poly(G·C)·poly(G·C)/CHE right, 1XAV/CHE in, 1XAV/CHE edge, 2HY9/CHE in, 2HY9/CHE edge, 143D/CHE in, 143D/CHE edge). The geometry of the ensemble was first minimized using molecular mechanics simulations by 4000 steps of steepest descent and 4000 steps of conjugated gradients algorithms in order to relax close contacts. Then the system is progressively heated until reaching 300K during 200 ps in the NVT ensemble, followed by 400 ps in the NPT ensemble to relax the global density of the water box. Finally production run has been performed for 100 ns in the NPT ensemble. To keep the temperature (300 K) and pressure (1 atm) constant, langevin dynamics and the Montecarlo thermostat have been used. All simulations were performed using periodic boundary conditions and the particle mesh Ewald for electrostatic interactions.

*Free energy calculation details:*

In practice, the free-energy differences between the different states were evaluated through a series of transformations between non-physical intermediate states (windows) connecting the initial to the final state by means of a coupling parameter l, varying from 0 to 1. All FEP and TI simulations were performed in both decoupling and coupling directions. For FEP simulations, the maximum-likelihood estimator of the free-energy difference was evaluated using data collected in both directions by means of the Bennett Acceptance Ratio (BAR) using the ParseFEP plugin [*J. Chem. Theory Comput.*, 2012, 8, 2606] of VMD. Both $\Delta G_{\text{alch}}^{\text{site}}$ and $\Delta G_{\text{alch}}^{\text{bulk}}$ FEP simulations were performed under the same conditions but using different stratification strategies tailored to ensure proper convergence of the free-energy estimates. Each window consists of 200 ps of equilibration followed by 800 ps of production. For $\Delta G_{\text{alch}}^{\text{site}}$ 60 windows were used for both coupling and uncoupling simulations. $\Delta G_{\text{alch}}^{\text{bulk}}$ was obtained by stratifying the coupling and uncoupling of CHE to the bulk in 55 windows. $\Delta G_{rest}^{site}$ and $\Delta G_{rest}^{bulk}$ were obtained by summing up the contributions of each individual restraint:

$$\Delta G_{rest}^{site} = \Delta G_{r}^{site} + \Delta G_{\theta}^{site} + \Delta G_{\phi}^{site} + \Delta G_{\Theta}^{site} + \Delta G_{\Phi}^{site} + \Delta G_{\Psi}^{site}$$

$$\Delta G_{rest}^{bulk} = \Delta G_{r,\theta,\phi}^{bulk} + \Delta G_{\Theta,\Phi,\Psi}^{bulk}$$

Contributions to $\Delta G_{\text{rest}}^{\text{site}}$ were determined through TI simulations stratified over 24 windows in both coupling and uncoupling directions. $\Delta G_{r,\theta,\phi}^{bulk}$ and $\Delta G_{\Theta,\Phi,\Psi}^{bulk}$ were calculated through numerical integration of analytical expressions [*Proc. Natl. Acad. Sci. U. S. A.,* 2005,102, 6825, *Biophys. J.*, 2001,81, 1632]. In the case of FEP simulations, a soft-core potential was employed in which the interatomic distance, r, used in the Lennard-Jones potential, was shifted $r \rightarrow r^2 + 5 \times (1-\lambda)$. Furthermore, in order to avoid the so-called ''end-point catastrophe'', electrostatic interactions of vanishing or appearing particles were linearly coupled to the simulation for l, varying from 0 to 0.5. At l values greater than 0.5, electrostatic interactions were fully decoupled from the simulation.

*Macromolecular ECD Modeling*

We employed the similar methodology as described in our previous works. [*Theor. Chem. Acc.*, 2015, 134, 36; *J. Phys. Chem. B*, 2016, 120, 3113; *J. Chem. Theory Comp, 2017*, 13, 3290] From the previously described molecular dynamics simulation, 100 structures have been extracted in order to represent the conformational space of each system. Then an ensemble of subunits is chosen in order to provide a suitable reproduction of the intrinsic circular dichroism of our system. Also based on our previous work [*J. Chem. Theory Comp, 2017*, 13, 3290], it appears that the best partitioning of the supramolecular chromophore is by achieved by decoupling it into 4 distinct chunks composed of three stacked nucleobases, as presented in Figure S12.

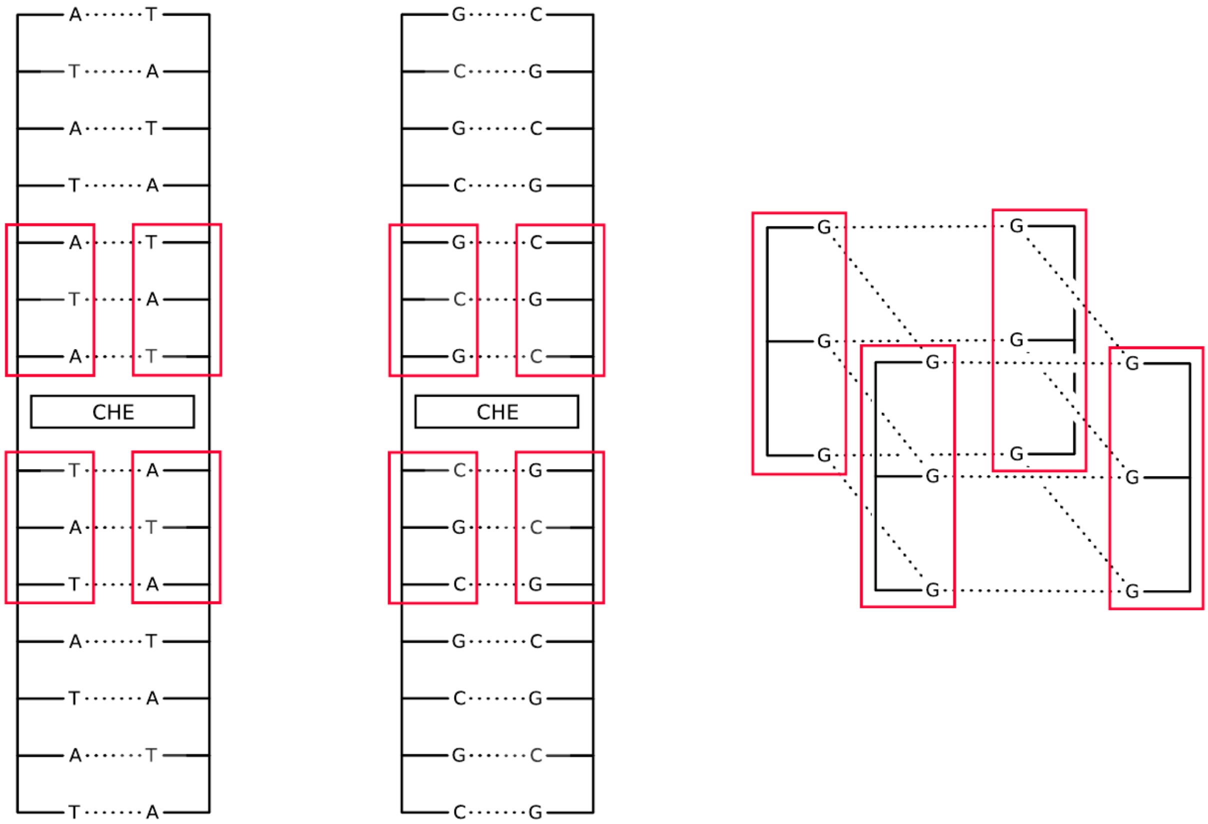


**Figure S12**. Partitioning of the macromolecular aggregate used to obtain the semiempirical Frenkel Hamiltonian in the case of B-DNA (left) and G4 (right). The red square represent the chunks included in the QM partition. Note that in all the case three stacked nucleobases are simultaneously included in each partition, hence allowing for the excited states delocalization over the whole subsystem.

Each subunit is described by QM/MM, including polarizable embedding, and covalent bonds are treated using the link atom method (hydrogen atom) to compute the first 12 excited states for each subunit at the TD-DFT M062X/6-31G(d,p) level of theory. The computations a locally have been realized using a modified version of the Gaussian09 code coupled with Tinker. From the QM/MM procedures the energies of the excited states as well as their transition dipole moments are obtained, hence allowing the definition of the Frenkel Hamiltonian whose diagonalization will then provide excitonic coupled states' energies and the corresponding circular dichroism rotational strength (intensities). The convolution of the Frenkel excitonic spectrum obtained for each snapshots will allow obtaining the final ECD spectrum. This has been obtained through Gaussian functions of fixed width at half-length (FWHL) of 0.4 eV.

## Components of the free energy calculations using the FEP approach

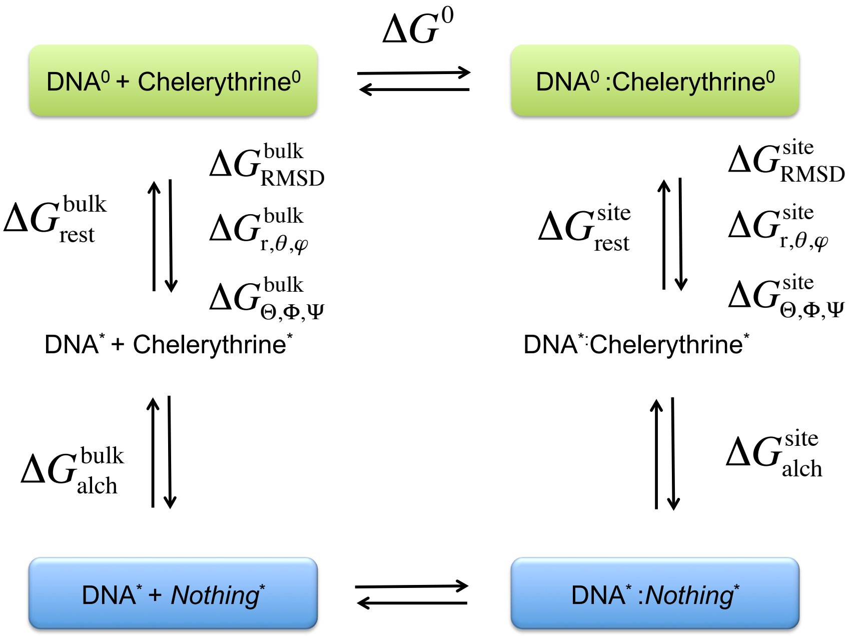


**Figure S13.** Thermodynamic cycle using for the calculation of the binding free energy. The free energy terms are indicated.

| Contributions | | Cmyc-edge Free energies (kcal/mol) | Cmyc-edge Time (ns) | Hybrid-in Free energies (kcal/mol) | Hybrid-in Time (ns) |
|---|---|---|---|---|---|
| $\Delta G_{rest}^{site}$ | $\Delta G_{RSMD}^{site}$ | -4.5 ± 0.5 | 96 | -4.6 ± 0.2 | 96 |
| | $\Delta G_{r}^{site}$ | 0 | | 0 | |
| | $\Delta G_{\theta}^{site}$ | -0.6 | | -0.3 | |
| | $\Delta G_{\varphi}^{site}$ | -0.7 | | -0.3 | |
| | $\Delta G_{\Theta}^{site}$ | -0.4 | | -0.2 | |
| | $\Delta G_{\Phi}^{site}$ | -0.3 | | -0.3 | |
| | $\Delta G_{\Psi}^{site}$ | -0.8 | | -0.6 | |
| $\Delta G_{alch}^{site}$ | | 53.5 ± 0.2 | 120 | 51.9 ± 0.3 | 120 |
| $\Delta G_{alch}^{bulk}$ | | -81.6 ± 0.2 | 110 | -81.6 ± 0.2 | 110 |
| $\Delta G_{rest}^{bulk}$ | $\Delta G_{RSMD}^{bulk}$ | 10.9 ± 0.4 | 96 | 12.2* | 96 |
| | $\Delta G_{r,\theta,\varphi}^{site}$ | 6.8 | | 6.6 | |
| | $\Delta G_{\Theta,\Phi,\Psi}^{site}$ | 4.2 | | 5.0 | |
| $\Delta G^{0}$ | | -13.5 | | -12.3 | |

[illegible]

**Table S1.** Calculated free energy components for the binding of CHELE to G4 DNA
